# An Unanticipated Diagnosis in a Child with Blunt Chest Trauma

**DOI:** 10.7759/cureus.96066

**Published:** 2025-11-04

**Authors:** Sri Hari Babu Sunkari, Nilesh Jagne, Soumya Ghoshal, Mahendra Chauhan

**Affiliations:** 1 Department of Trauma and Emergency, All India Institute of Medical Sciences, Nagpur, Nagpur, IND

**Keywords:** blunt chest trauma, hemopneumothorax, malignant mesenchymal neoplasm, oncology, thoracotomy

## Abstract

A 13-year-old girl presented 20 days after blunt chest trauma with persistent hemopneumothorax despite intercostal drainage (ICD). Imaging revealed a large peripherally enhancing lesion in the right chest with a third rib fracture, raising suspicion of retained hemothorax. She underwent thoracotomy and decortication, and histopathology identified a malignant mesenchymal neoplasm with pulmonary metastasis. This case highlights the importance of considering malignancy in trauma patients whose clinical course deviates from expectations and demonstrates the diagnostic value of early advanced imaging and surgical exploration.

## Introduction

Blunt chest trauma is one of the most common emergencies encountered in trauma care. Typical consequences include rib fractures, pneumothorax, and hemothorax, which usually resolve with timely tube thoracostomy or surgical management [1]. Rarely, underlying pathologies such as malignancy can complicate the clinical picture and delay diagnosis [2]. Malignant mesenchymal neoplasms of the thorax are uncommon, particularly in children, and may mimic trauma-related injuries on imaging [3, 4]. Their rarity and nonspecific features make diagnosis challenging in the emergency setting. This case describes how an occult malignancy presented as a massive hemothorax in a child following trivial trauma, emphasizing the importance of maintaining a broad differential diagnosis when clinical progression deviates from expectations.

## Case presentation

A 13-year-old girl presented 20 days after a trivial fall with blunt chest trauma. At the referring hospital, a right intercostal drainage (ICD) tube was inserted for presumed hemopneumothorax, but her symptoms persisted, prompting transfer. On arrival, she was alert with a Glasgow Coma Scale score of 15/15 [5]. She was tachypneic with a respiratory rate of 30 breaths per minute, tachycardic at 160 beats per minute, and with blood pressure 90/56 mmHg. Her oxygen saturation was 95% on room air. The patient was previously healthy, with no history of chronic illness, prior hospitalizations, or respiratory symptoms before the trauma event. She had no known bleeding diathesis, coagulopathy, or other medical comorbidities and was not on any regular medications. Examination revealed tracheal deviation to the left, diminished right chest expansion, markedly reduced breath sounds, and dullness to percussion. The ICD column was static, suggesting poor drainage. No external injuries, pelvic instability, or limb fractures were identified. A provisional diagnosis of blunt chest trauma with right-sided massive hemopneumothorax and ineffective ICD drainage was made.

Investigations

Baseline hematology, including complete blood count and coagulation profile, was normal (Table 1).

**Table 1:** Baseline hematological investigations with reference ranges PT: prothrombin time; aPTT: activated partial thromboplastin time; INR: international normalized ratio

Parameter	Result	Reference range
Hemoglobin	12.4 gm/dl	11.5 to 15 gm/dl
Total leukocyte count	10,900/cumm	4000 to 11,000/cumm
Platelet count	4.35 x 10^5 ^/cumm	1.5 to 4.5 x 10^5 ^/cumm
PT	14.4 seconds	14.09 – 16.33 seconds
aPTT	28 seconds	25 – 35 seconds
INR	1.07	0.8 – 1.5

**Figure 1:**
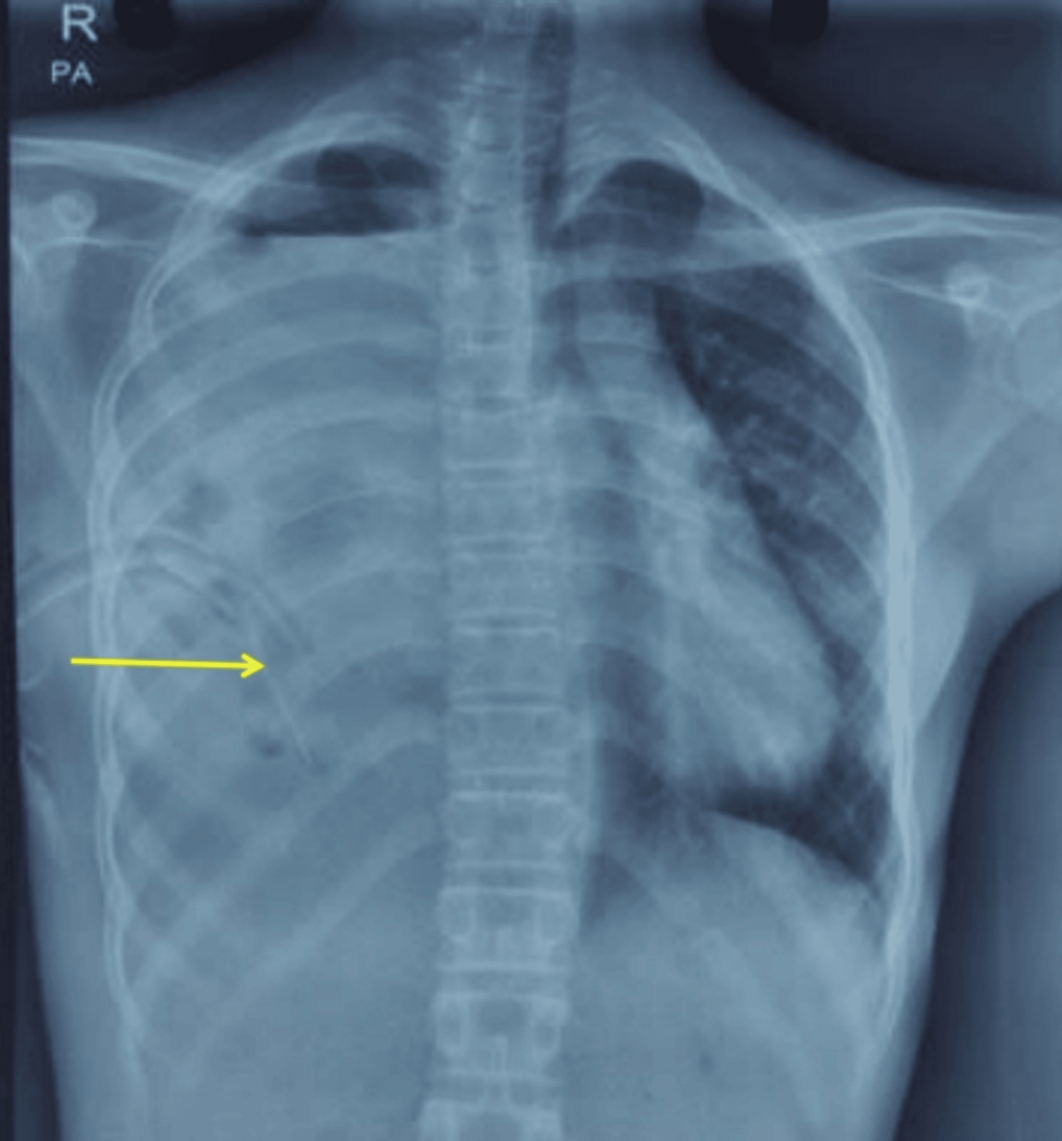
Initial chest X ray (PA) showing right-sided hemopneumothorax with intercostal drainage tube in situ (arrow). PA: posteroanterior

Chest radiograph confirmed a massive right hemopneumothorax with the ICD in situ (Figure 1).

**Figure 2:**
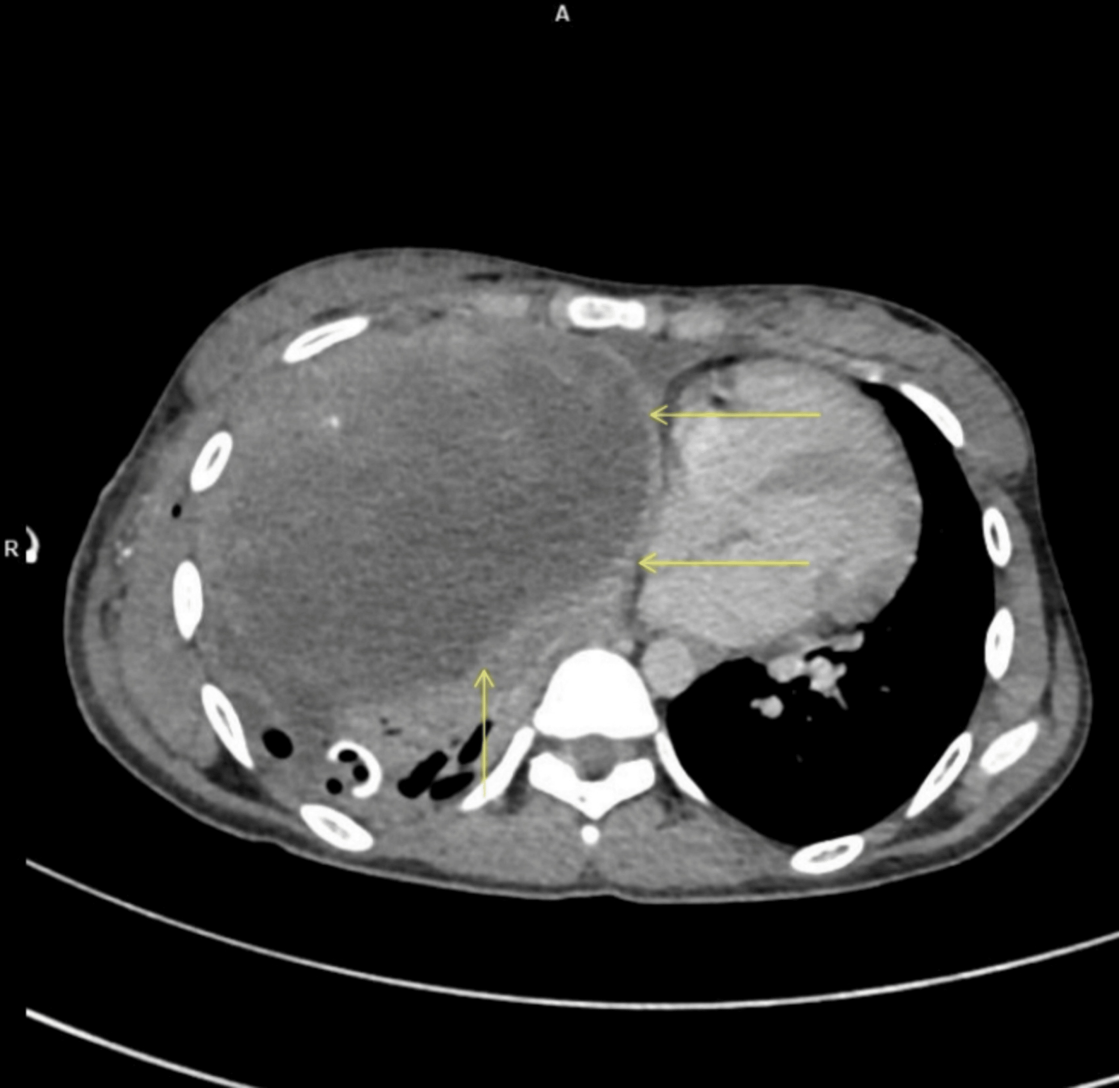
Contrast-enhanced CT thorax (axial view) demonstrating a peripherally enhancing lesion in the right hemithorax (arrows).

Contrast-enhanced computed tomography (CECT) of the thorax showed a large, peripherally enhancing lesion in the right lung (Figure 2), associated hemopneumothorax, and a right third rib fracture. No intercostal vascular injury or diaphragmatic rupture was identified.

Differential diagnosis

Traumatic hemopneumothorax with rib fracture was common after blunt trauma, supported by initial radiographic findings. Pulmonary contusion was considered but less likely, as the radiologic progression was atypical. Infective collection or empyema was suggested by persistent symptoms, but the absence of fever or elevated total leukocyte count made this less likely. Intercostal vessel injury and diaphragmatic rupture were considered but excluded on CT imaging. Underlying malignancy was considered due to the unusually large peripherally enhancing lesion in the right lung, persistent opacification despite drainage, and poor clinical recovery.

Treatment

Initial management followed Advanced Trauma Life Support principles [6] with airway stabilization, oxygen, fluids, and analgesia. The existing ICD was repositioned, but no column movement. A second ICD was inserted at a higher level, draining only 100 mL of fluid. Column movement ceased after one hour, suggesting a loculated or organized collection.

**Figure 3:**
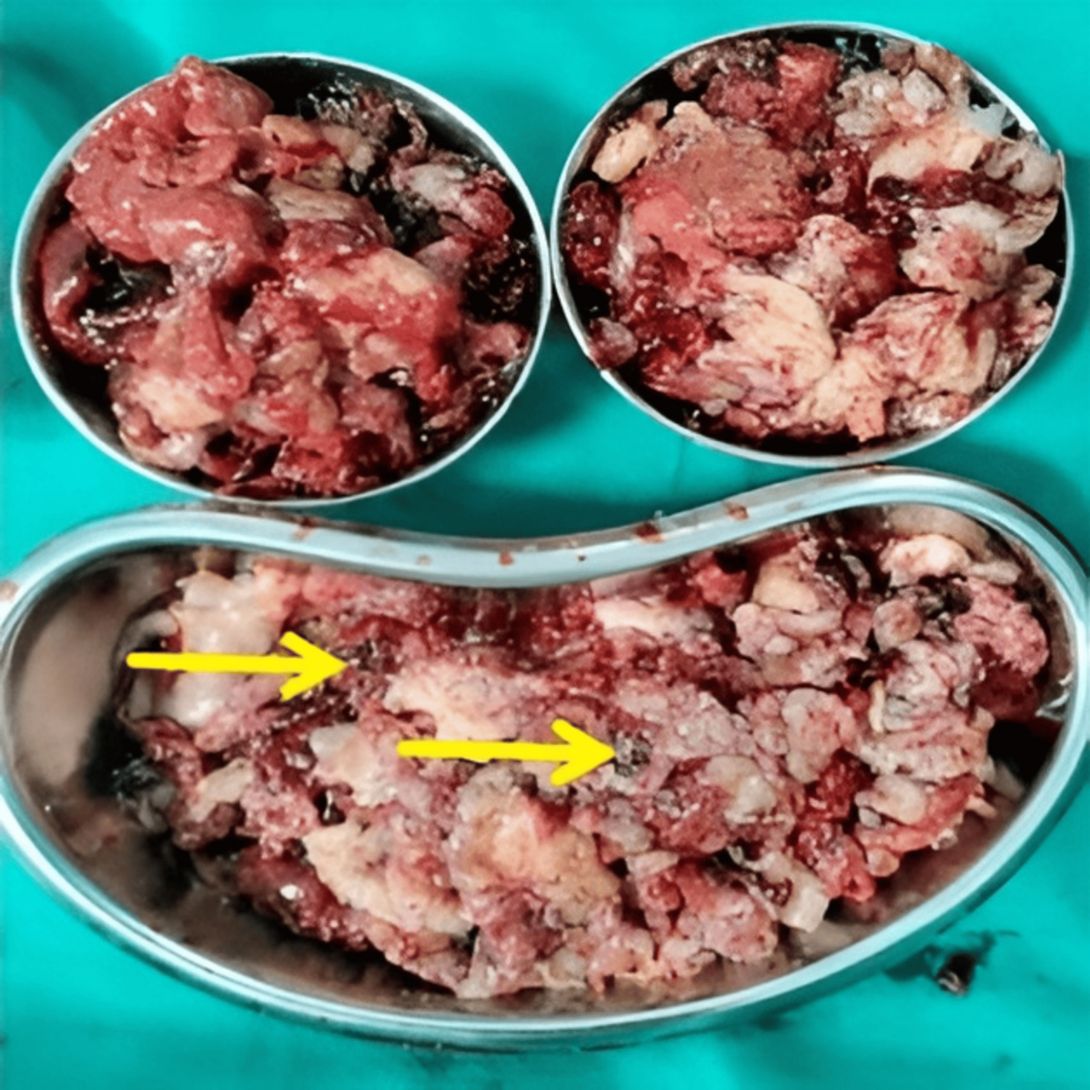
Decortication specimen revealing greyish irregular tissue fragments with hemorrhagic areas (arrows).

In view of persistent symptoms, thoracic surgery was undertaken. Open thoracotomy with decortication was performed, evacuating a greyish, irregular tissue with hemorrhagic areas (Figure 3). The specimen was submitted for histopathological examination.

**Figure 4:**
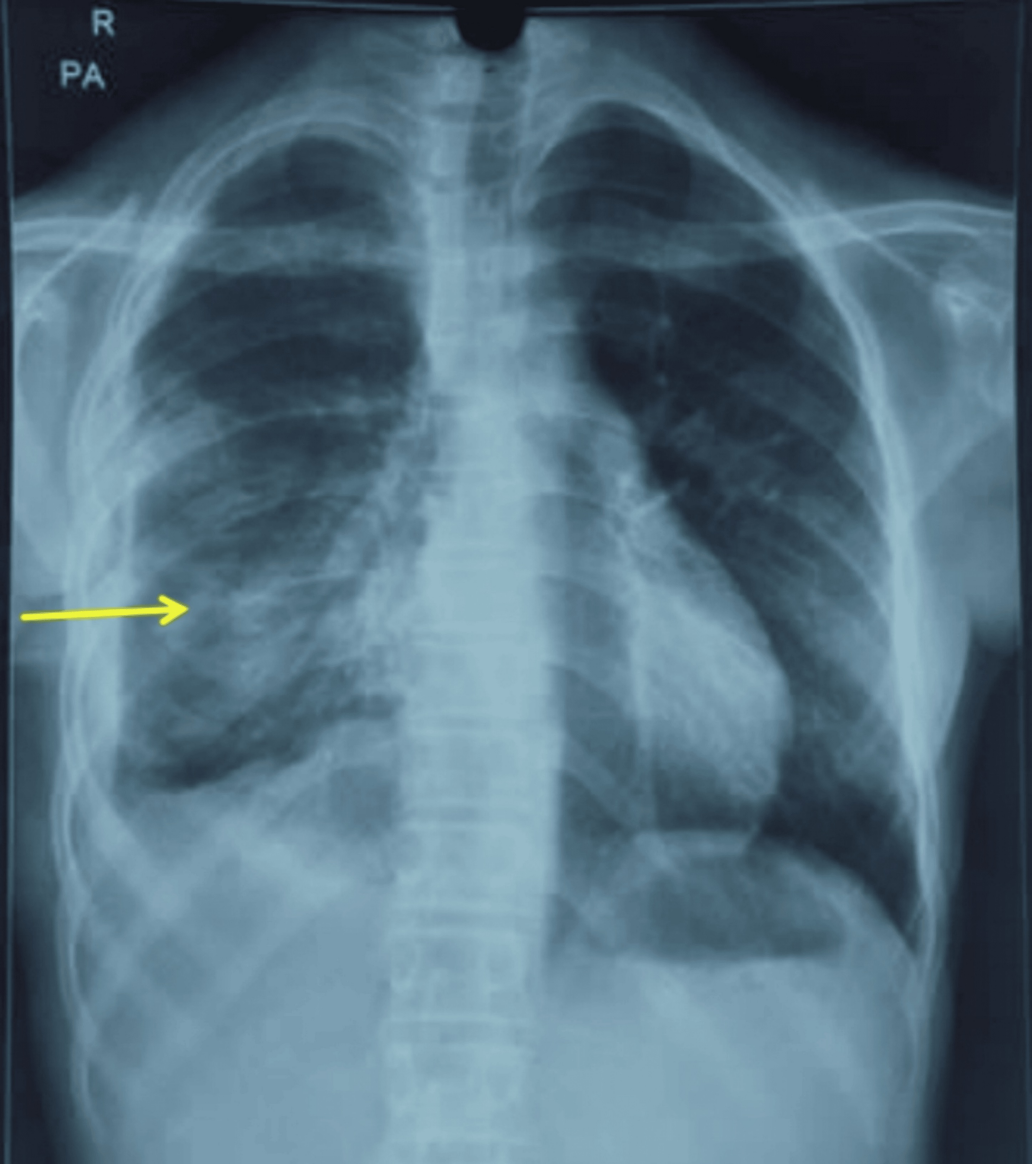
Post-decortication chest X ray (PA) demonstrating near-complete right lung expansion (arrow). PA: posteroanterior

Postoperatively, she was monitored in intensive care with oxygen, antibiotics, analgesia, and incentive spirometry. Serial chest radiographs confirmed satisfactory lung re-expansion (Figure 4).

Outcome and follow-up

**Figure 5:**
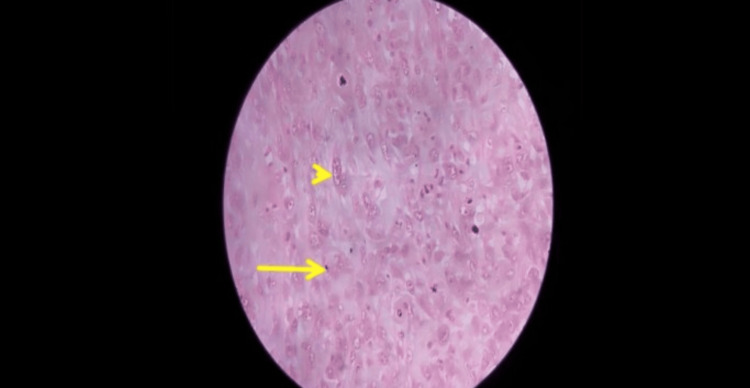
Histopathological section showing large tumor cells with hyperchromatic pleomorphic nuclei and of places showing prominent nucleoli (arrow head). Mitotic activity was increased (arrow).

The patient improved and was discharged on day 15 post surgery. Histopathology revealed a malignant mesenchymal neoplasm (Figure 5).

**Figure 6:**
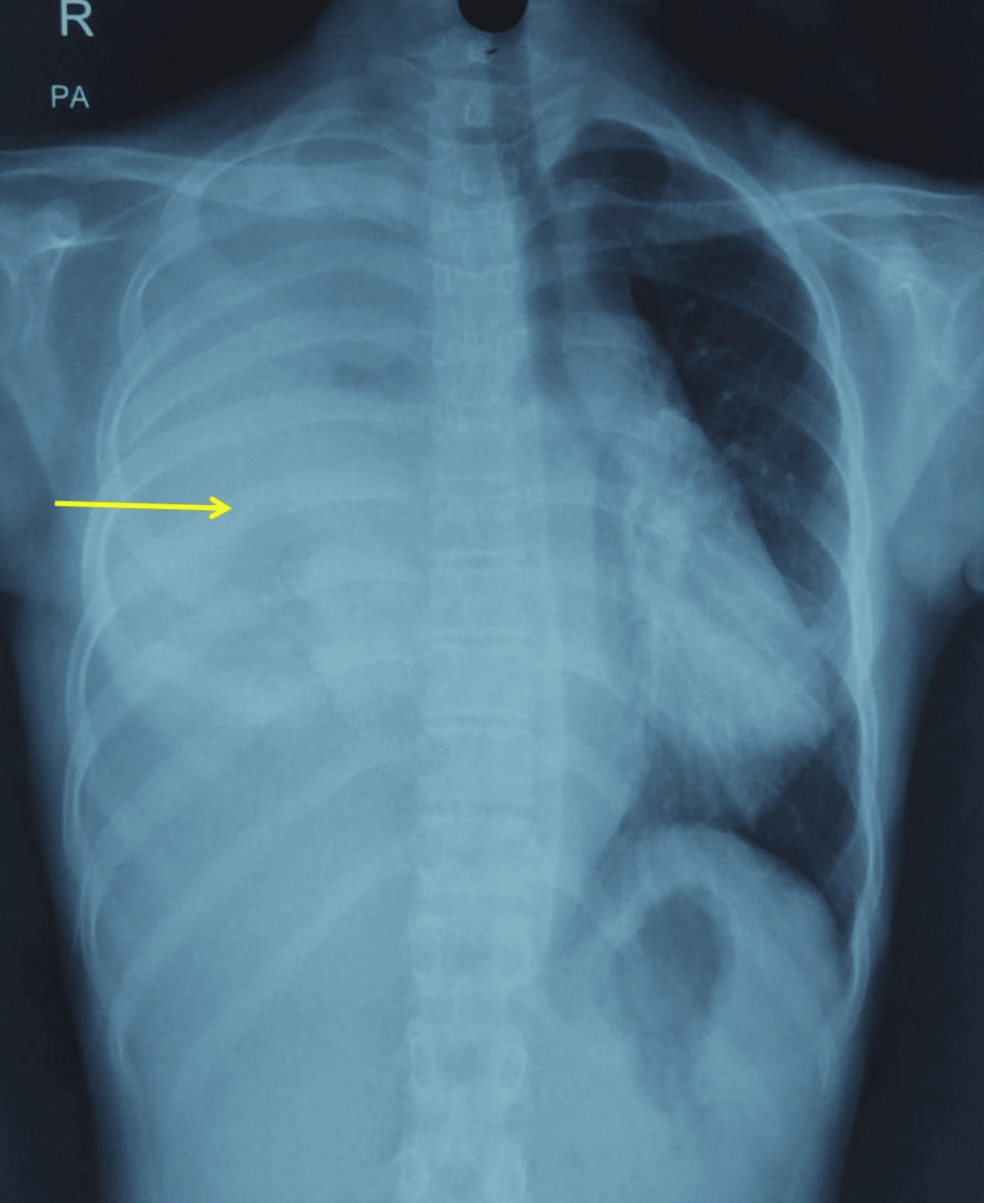
Follow-up chest X ray(PA) showing right-sided white-out lung (arrow) with mediastinal shift towards the left. PA: posteroanterior

A follow-up chest radiograph showed a white-out right lung with mediastinal shift to the left (Figure 6).

PET-CT demonstrated contralateral pulmonary metastasis with no extra-thoracic disease. She was referred to medical oncology and commenced systemic chemotherapy. At reporting, she has completed three cycles and remains under follow-up.

## Discussion

Malignant mesenchymal tumors of the thorax are exceedingly uncommon, especially in children, and may imitate more common traumatic conditions. In trauma patients, hemothorax is usually due to rib fracture, pulmonary laceration, or vascular injury [1]. Persistent collections that fail to resolve after appropriate tube thoracostomy warrant further evaluation. Organized hematomas, empyema, and rare intrathoracic malignancies can mimic hemothorax [2].

The management of typical traumatic hemothorax differs significantly from that of malignancy-associated hemothorax. In usual traumatic hemothorax, surgical intervention is generally indicated when >1500 mL of blood is evacuated immediately after tube thoracostomy, when chest-tube drainage persists at 150-200 mL/h for two to four hours, or when continuous transfusion is required to maintain hemodynamic stability [1]. Otherwise, tube thoracostomy, resuscitation, and supportive care are usually sufficient. When an underlying neoplastic process is suspected, however, obtaining an image-guided or thoracoscopic biopsy is ideal, as it can confirm the diagnosis and help avoid unnecessary extensive surgery [3]. Once malignancy is established histologically, early staging with PET-CT and prompt medical-oncology involvement enable initiation of systemic therapy with reduced operative morbidity.

In this case, biopsy was not initially considered because CECT of the thorax demonstrated a peripherally enhancing collection that, in the clinical context of recent trauma and an associated rib fracture, appeared consistent with a retained post-traumatic hemothorax. Given the patient’s hemodynamic instability and persistent collection, surgical decortication was pursued to achieve immediate decompression and stabilization. The unexpected histopathological diagnosis of malignant mesenchymal neoplasm and subsequent recurrence on follow-up highlight the importance of maintaining a differential diagnosis that includes occult malignancy when the clinical course deviates from expected recovery. Advanced imaging and surgical exploration ultimately enabled tissue diagnosis, guiding appropriate oncologic management.

Primary pulmonary sarcomas and other mesenchymal neoplasms constitute less than 0.5% of lung tumors [7]. Pediatric cases are rare but reported. Kadabur et al. described three cases of primary pulmonary rhabdomyosarcoma in children [4]. Bayan Hafiz et al. reported pediatric alveolar rhabdomyosarcoma initially mistaken for pneumonia [8]. Similarly, pleural synovial sarcomas and inflammatory myofibroblastic tumors can present with non-specific symptoms and radiographic opacities [3, 9]. Spontaneous hemothorax secondary to intrathoracic malignancy, although rare, has been documented in lung carcinoma and mesothelioma-like presentations [10, 11].

Certain practices should be avoided in the emergency setting when encountering similar cases. Repeated blind repositioning of chest tubes, aggressive negative suction, or forceful flushing of a non-draining ICD should be avoided, as these maneuvers may exacerbate bleeding from friable tissue and delay diagnosis. Instead, clinicians should consider targeted imaging, early image-guided biopsy when feasible, and multidisciplinary consultation whenever drainage output is minimal or radiographic opacities persist [11].

This case emphasizes the need for a high index of suspicion and multidisciplinary care, including early trauma surgery and oncology involvement.

## Conclusions

Trauma can sometimes mask rare but aggressive intrathoracic malignancies. In patients whose recovery does not follow the expected trajectory, such as those with persistent opacities, minimal ICD drainage, or recurrent collections, clinicians should maintain a broad differential diagnosis that includes malignancy. Unnecessary procedures such as repeated tube repositioning or aggressive suctioning should be avoided, as they may worsen bleeding and delay diagnosis. Early use of advanced imaging, consideration of image-guided biopsy, and timely multidisciplinary involvement can help identify atypical causes early, optimize patient outcomes, and prevent avoidable surgical interventions.

## References

[1] Karmy-Jones R, Jurkovich GJ (2004). Blunt chest trauma. Curr Probl Surg.

[2] Ghaffar A, Gillani SA, Imam SF (2023). Unmasking the unforeseen: A case of radiological tension empyema mimicking a traumatic hemothorax. Cureus.

[3] Xu Y, Lin J, Sun H, Xie S (2020). Primary pleural synovial sarcoma in an adolescent: a case report. Transl Cancer Res.

[4] Lokesh KN, Premalata CS, Aruna Kumari BS, Appaji L (2013). Primary pulmonary rhabdomyosarcoma in children: report of three cases with review of literature. Indian J Med Paediatr Oncol.

[5] Teasdale G, Jennett B (1974). Assessment of coma and impaired consciousness. A practical scale. Lancet.

[6] (2023). Advanced Trauma Life Support® program—ATLS® 11. https://www.facs.org/quality-programs/trauma/education/advanced-trauma-life-support/atls-11/?utm_source=chatgpt.com.

[7] Travis WD, Brambilla E, Nicholson AG (2015). The 2015 World Health Organization classification of lung tumors: impact of genetic, clinical and radiologic advances since the 2004 classification. J Thorac Oncol.

[8] Hafiz B, Bamefleh H (2022). Primary pulmonary alveolar rhabdomyosarcoma in a pediatric patient: a case report with literature review. Cureus.

[9] Seyedi SJ, Saeidinia A, Dehghanian P (2022). Pulmonary inflammatory myofibroblastic tumor in a male child: a case report. Clin Case Rep.

[10] Snaebjornsson P, Vos CG, Hartemink KJ, Lely RJ, Samii SM, Grünberg K, Paul MA (2011). Fatal hemothorax caused by pseudomesotheliomatous carcinoma of the lung. Patholog Res Int.

[11] Wethasinghe J, Sood J, Walmsley R, Milne D, Jafer A, Gordon-Glassford N (2015). Primary pleural epithelioid hemangioendothelioma mimicking as a posterior mediastinal tumor. Respirol Case Rep.

